# Crambescidin 800, Isolated from the Marine Sponge *Monanchora viridis*, Induces Cell Cycle Arrest and Apoptosis in Triple-Negative Breast Cancer Cells

**DOI:** 10.3390/md16020053

**Published:** 2018-02-08

**Authors:** Sumi Shrestha, Anabel Sorolla, Jane Fromont, Pilar Blancafort, Gavin R. Flematti

**Affiliations:** 1 School of Molecular Sciences, The University of Western Australia, Crawley 6009, Western Australia, Australia; sumi.shrestha@research.uwa.edu.au; 2 Cancer Epigenetics, Harry Perkins Institute of Medical Research, QEII Medical Centre and Centre for Medical Research, The University of Western Australia, Crawley 6009, Western Australia, Australia; anabel.sorollabardaji@uwa.edu.au; 3 Western Australian Museum, Welshpool 6106, Western Australia, Australia; jane.fromont@museum.wa.gov.au

**Keywords:** crambescidin 800, triple-negative breast cancer, marine sponge

## Abstract

Triple negative breast cancer (TNBC) is currently the only group of breast cancers without an effective targeted therapy. Marine sponges have historically been a source of compounds with anticancer activity. In this study, we screened extracts from twenty marine sponges collected off the coast of Western Australia for cytotoxic activity against TNBC cells. One very active extract derived from the sponge *Monanchora viridis* was selected for bioactivity-guided fractionation. Through multiple steps of purification, we isolated a potent cytotoxic compound, which was identified as crambescidin 800 (C800). We found that C800 exhibited cytotoxic potency in a panel of breast cancer cells, of which TNBC and luminal cancer cell models were the most sensitive. In addition, C800 induced cell cycle arrest at the G2/M phase, resulting in a decline in the expression of cyclin D1, CDK4, and CDK6 in TNBC cells. This effect was associated with the inhibition of phosphorylation of Akt, NF-κB, and MAPK pathways, resulting in apoptosis in TNBC cells.

## 1. Introduction

Breast cancer is the most common malignancy among women worldwide [1,2]. Among various subtypes of breast cancer (Luminal A/B, basal-like and human epidermal growth receptor 2 (HER2) enriched) identified by transcriptomic analyses of breast tumors, basal-like breast cancer is one of the most aggressive subtypes [3,4,5]. Basal-like breast cancers comprise a large fraction (~80%) of triple negative breast cancers (TNBC), which lack an estrogen receptor (ER), a progesterone receptor (PR), and HER2 [4]. Thus, these breast cancers do not have any targeted therapies available, unlike other breast cancer subtypes. Although TNBCs represent ~15% of all breast cancers, these tumors are characterised by poor differentiation, high proliferation, and metastatic behavior [6]. The treatment of choice for TNBC is standard chemotherapy, such as administration of taxanes (e.g., docetaxel), platinum agents (e.g., cisplatin), anthracyclines (e.g., doxorubicin), and antimetabolites (e.g., 5-fluorouracil) [7,8]. However, these chemotherapeutic drugs cause a number of side effects [8]. In addition, acquired resistance mechanisms almost invariably occur in the metastatic setting, rendering these drugs therapeutically ineffective [7]. Therefore, there is an urgent need to find novel treatments to combat this deadly disease.

Natural products play an important role in pharmacological research and are an important source of lead compounds for the development of drugs [9]. It is estimated that about 40% of all medicines are natural products or semi-synthetic derivatives of natural products [10,11]. Marine natural products in particular have inspired researchers to identify novel compounds that could eventually lead to therapeutics [12]. Marine organisms, such as sponges, live in competitive environments and to protect themselves from predation and overgrowth, produce compounds with defensive characteristics. Such compounds have been shown to have cytotoxic activity and, as such, are of interest in pharmaceutical research [13]. Cytarabine (Ara C) is an anticancer drug that was developed from a nucleoside, originally isolated from a Caribbean sponge *Tectitethya crypta* (formerly known as *Cryptotethya crypta*) [13,14]. Similarly, the recently developed drug Eribulin mesylate, which is used for the treatment of metastatic breast cancer [15], is a simpler synthetic analogue of halichondrin B isolated from the sponge *Halichondria okadai* [16]. Hence, marine sponges are a rich source of compounds with potential anticancer activities. 

**Figure 1:**
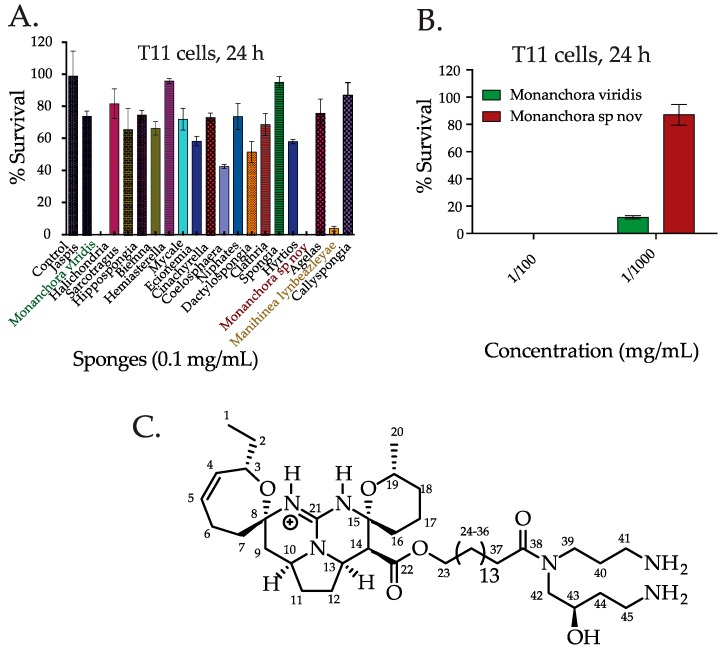
Screening of anticancer activity of crude solvent extracts of twenty marine sponges collected in WA and chemical structure of the bioactive compound isolated from the sponge *Monanchora viridis*. (**A**) Percentage of cell survival after 24 h of treatment with crude solvent extracts of 20 sponges in T11 cells. Cell survival assay was performed using CellTiter-Glo^®^ and measured as the percentage of cell survival compared to vehicle control (0.1% DMSO diluted in media). The active sponges in T11 cells were *Monanchora viridis*, *Monanchora* sp. nov*.,* and *Manihinea lynbeazleyae*, which are highlighted in green, red, and orange, respectively. (**B**) Comparison between the percentage of cell survival in T11 cells when treated with the active sponge extracts from *Monanchora viridis* and *Monanchora* sp. nov. at different dilutions after 24 h. (**C**) Chemical structure of crambescidin 800, which was isolated as the bioactive compound from *Monanchora viridis*.

There has been a wealth of research into the chemistry of marine sponges collected off the coast of the America’s, South East Asia, Japan, and Eastern Australia [17]. Reports show that in the period 2001–2010, the contribution of Japanese sponge samples to novel compounds was the highest (20 compounds each year), followed by Indonesia, Korea, Australia, China, and Papua New Guinea [17]. Despite the studies on Eastern Australian marine species [18], the chemistry of Western Australian marine sponges remains relatively underexplored [19,20]. In this study, we investigated the cytotoxic activity against TNBC cells with crude solvent extracts of sponges collected off the coast of Western Australia and stored in the Western Australian Marine Bioresources Library (WAMBL). The WAMBL represents a unique resource of biodiversity, with many of the sponges currently only classified to genus. Of the twenty sponges that we initially tested, three sponges of the genera *Manihinea* and *Monanchora* showed cytotoxic activity in TNBC cells. Of the three active sponges, two sponges belonging to the same genus *Monanchora* were the most active (Figure 1A). Following bioassay-guided fractionation of the most active sponge *Monanchora viridis*, we isolated crambescidin 800 (C800), a guanidine alkaloid, as an active compound against TNBC cells. Guanidine alkaloids from sponges of genus *Monanchora* are reported to have diverse chemical structures and biological activities [21,22,23,24], which includes inducing cytotoxic activities [25,26] and apoptosis against leukemia cells [25,27], overcoming drug resistance by induction of autophagy and lysosomal membrane permeabilisation in urogenital cancer cells [28], and inhibiting HIV-1 fusion [29], transient receptors potential channels [30], and EGF-induced neoplastic transformation in cancer cells [31]. It was also reported that the alteration of the compound’s structure affected its cytotoxic activities, inducing apoptosis, cell cycle progression, and the induction of signaling pathways in cancer cells [31].

In this study, using C800, we performed a wide range of biological assays such as cell viability, cell cycle analysis, and apoptotic cell death and probed the signaling pathways involved in its mechanism of action. We showed that C800 exhibited cytotoxic activity in a panel of breast cancer cell lines, with TNBC cells showing more significant differences in cell viability than immortalized fibroblasts. We compared the cytotoxic potency of C800 with chemotherapeutic drugs and found that C800 has low micromolar potency, a level of potency seen with cisplatin and 5-fluorouracil, which are clinically used as chemotherapeutic drugs for treating TNBC. In addition, we studied the cell cycle analysis and the signaling pathways triggered by C800 in TNBC cells and found that its cytotoxic effect is caused by cell cycle arrest at the G2/M phase, inhibition of cyclin D1, CDK4, and CDK6, and inhibition of phosphorylation of Akt/mTOR, NF-κB, and MAPK pathways, leading to apoptosis in TNBC cells.

## 2. Results and Discussion

### 2.1. Screening the Extracts from Western Australian (WA) Marine Sponge in TNBC Cells and Bioassay Guided Isolation of C800

We screened the cytotoxic activity of the crude solvent extracts from twenty marine sponges collected from WA in TNBC cells by a CellTiter Glo assay, which monitors mitochondrial ATP release and thus measures cell viability. TNBC models that were used in this study (T11 and SUM159PT) are mesenchymal cells stem, cell-enriched, highly migratory, and have low expression of claudin and junction proteins, making these cells highly resistant to drugs [4]. In the first round of screening, we found that the extracts belonging to the genera *Manihinea* and *Monanchora* (*Monanchora* represented by two species, *Monanchora viridis* and *Monanchora* sp. nov.) were active in TNBC murine claudin-low T11 cells derived from p53 ^−^/^−^ transgenic mice [4,32,33] (Figure 1A). Thus, T11 cells can recapitulate faithfully the characteristics of a TNBC model. As the extracts from the *Monanchora* sponges were the most active, these sponges were investigated further in this study. At low dilution (ca. 0.01 mg extract/mL), both the *Monanchora* extracts were equally active while at higher dilution (0.001 mg/mL), *M. viridis* was 10 times more active than *M.* sp. nov. in T11 cells (Figure 1B). Hence, for further fractionation and isolation of the active compound, *M. viridis* was selected. The methanol extract of *M. viridis* was sub-fractionated initially by solvent partitioning between water and dichloromethane (DCM). The active DCM fraction was separated by flash chromatography on a C18 reversed phase silica column using gradient elution starting from 100% water to 100% methanol to obtain six fractions. The 80% methanol fraction was the most active in T11 cells and this fraction was further separated by high performance liquid chromatography (HPLC) using an isocratic solvent system of 45% (*v*/*v*) acetonitrile/water (+0.1% TFA). A total of 15 fractions were collected and tested on T11 cells. The most active fraction was found to contain one major compound of sufficient purity (>95%) to enable structural elucidation to be attempted. Using high-resolution mass spectrometry, the protonated molecular ion was found to be *m*/*z* 801.6112, corresponding to a molecular formulae of C_45_H_80_N_6_O_6_. By analyzing 1D and 2D NMR spectroscopy techniques and comparing the data with known secondary metabolites from other *Monanchora* species [34,35], we identified the active compound as crambescidin 800 (C800, Figure 1C). HPLC-MS analysis of the crude solvent extract of the other active *Monanchora* sponge, *M.* sp. nov., showed that C800 was also present in the extract and assumed to be responsible for its activity. Quantification of the extract by LC-MS showed that the concentration of C800 was much lower in *M.* sp. nov., which was consistent with the greater activity of *M. viridis* compared to *M.* sp. nov. in T11 cells. 

### 2.2. C800 Decreases Cell Viability in a Panel of Breast Cancer Cells

**Figure 2:**
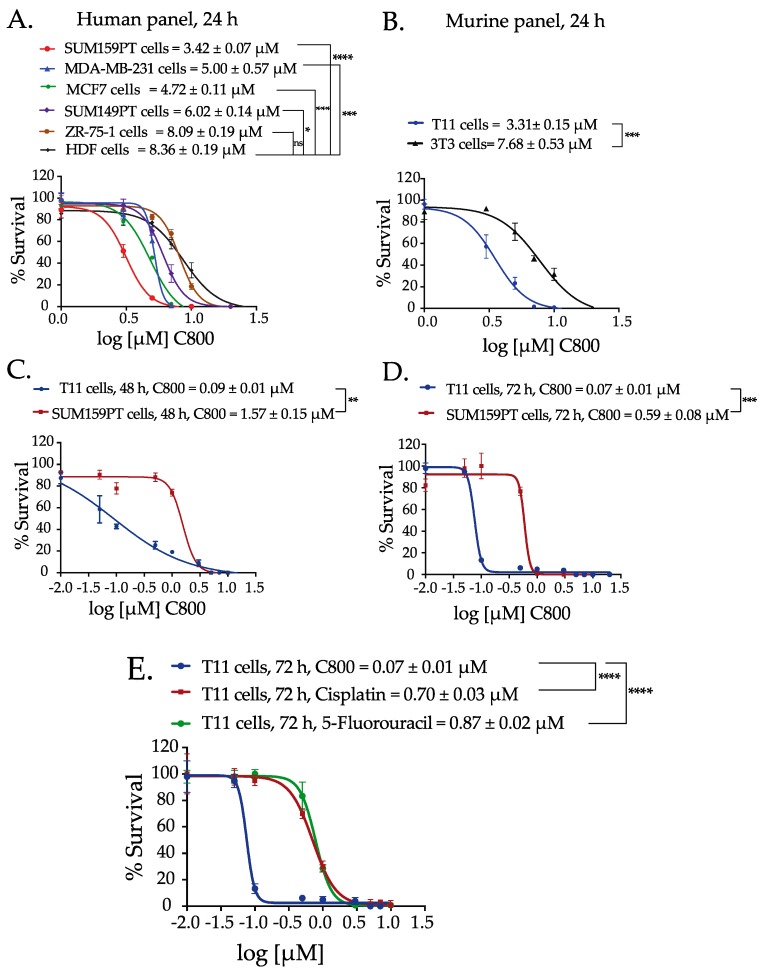
C800 inhibits cell survival in a panel of breast cancer cells. (**A**) Dose response curve for cytotoxic effect of C800 in a panel of human breast cancer (TNBC and luminal) and immortalized fibroblasts after 24 h of treatment. (**B**) Dose response curve for cytotoxic effect of C800 in murine TNBC cell line, T11, and immortalized fibroblasts, 3T3 after treatment for 24 h. Dose response curve for cytotoxic effect of C800 in T11 and SUM159PT cells after (**C**) 48 h and (**D**) 72 h of treatment. (**E**) Comparison between the dose-response curves of C800 with currently used chemotherapeutic drugs, cisplatin, and 5-fluorouracil on T11 cells after 72 h of continuous exposure. T11 data is the same as in graph (**D**). Each of these experiments was done in triplicate, and repeated three times. Results were plotted in Graphpad Prism 6; the graphs are selected ‘representative’ graphs and the values above the graphs are the IC_50_ ± SD from three independent experiments. Statistical analysis was performed with one way ANOVA with Tukey’s posthoc test **** *p* < 0.0001, *** *p* < 0.001, ** *p* < 0.01, * *p* < 0.1, ns = not significant.

Next, we evaluated the effect of C800 in reducing the cell viability in a panel of breast cancer cell lines, which includes claudin-low TNBC cells (T11, SUM159PT and MDA-MB-231), basal-like TNBC cells (SUM149PT), luminal cells (MCF7 and ZR-75-1), and immortalized fibroblasts (3T3 and HDF). We observed that C800 after 24 h of treatment inhibited the viability of cells at concentrations ranging from 3.31 to 8.36 µM (Figure 2A,B). Interestingly, C800 was effective in each of the breast cancer cell lines tested. However, there was a significant difference in cytotoxic potency in TNBC cells (T11 and SUM159PT) compared to immortalized fibroblasts (3T3 and HDF). The IC_50_ of C800 in immortalized fibroblasts (3T3 and HDF) were twice that required to inhibit the survival of TNBC cells (T11 and SUM159PT) (Figure 2A,B). As T11 and SUM159PT cells were most sensitive to C800, these cells were chosen to further investigate the time dependence of C800 in decreasing the viability of breast cancer cells. Cells were treated with different concentrations of C800 for 24, 48, and 72 h. The IC_50_ of C800 in T11 cells when treated for different times reduced drastically from 3.31 µM at 24 h to 0.09 µM at 48 h, and to 0.07 µM at 72 h. In SUM159PT cells, the IC_50_ of C800 also reduced from 3.42 µM at 24 h to 1.57 µM at 48 h, and to 0.59 µM at 72 h (Figure 2C,D). This result indicates that the effect of C800 in these cells is time dependent. Further investigation of the mechanism of action of C800 was carried out on T11 and SUM159PT cells. For all further experiments, two sets of concentrations were used; concentrations lower than IC_50_ (0, 0.5, and 1 µM) and concentrations equal to and higher than the measured IC_50_ (5, 10, and 20 µM).

We also compared the effect of C800 on cell viability with that of conventional chemotherapeutic drugs used in the clinic to treat TNBC, such as cisplatin and 5-fluorouracil. As T11 showed most sensitivity toward C800 at 72 h, the studies were performed in T11 cells after 72 h of treatment. Interestingly, C800 exhibited significantly higher potency in T11 cells compared with both chemotherapy agents. Specifically, the IC_50_ of C800 in T11 cells was found to be 0.07 µM (70 nM), whereas for cisplatin, the IC_50_ was 0.70 µM and 0.87 µM for 5-Fluorouracil. Overall, these data indicate that C800 exhibits nanomolar potency and 10-fold more activity compared with current chemotherapeutic drugs used clinically to treat TNBC. 

C800 is a guanidine alkaloid isolated mostly from sponges such as *Crambe crambe* [34,36,37] and *Monanchora* sp [38,39] and has been demonstrated to have diverse biological activities [38,40]. It has been found that C800 induces erythroid differentiation in chronic leukemia cells (K562 cells), leading to cell cycle arrest at S-phase and promotes neurite outgrowth in Neuro-2A neuroblastoma cells [41]. Also, it has been shown to induce dose-dependent antioxidant activity against glutamate-induced oxidative stress in hippocampal cells in mice [42]. Similarly, another analogue of C800 from *Crambe crambe*, C816, has shown activity against liver-derived tumor cells inhibiting cell cycle progression in the G0/G1 phase, cell migration, and cell-matrix adhesion [43]. However, the effects of crambescidins or similar compounds have not yet been explored in breast cancer, particularly for highly proliferative TNBC models, which we investigate further below. 

### 2.3. C800 Induces Cell Cycle Arrest in TNBC

**Figure 3:**
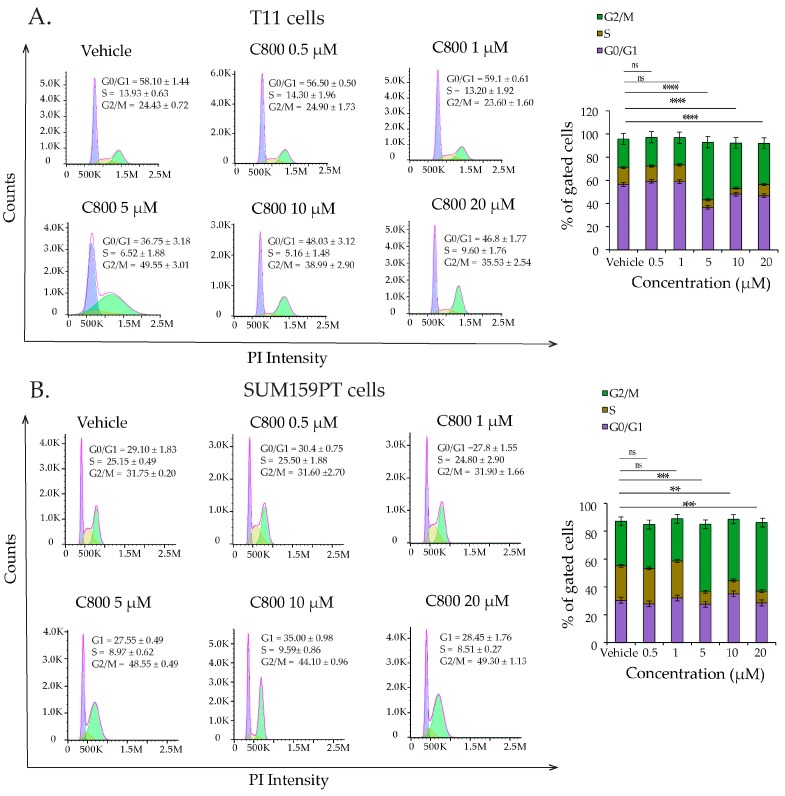
C800 induces cell cycle arrest in TNBC cell lines. (**A**) Effect of C800 on cell cycle distribution in T11 cells after 24 h of treatment. (**B**) Cell cycle analysis of SUM159PT cells when treated with C800 for 24 h. Cells were treated with 0.5, 1, 5, 10, and 20 μM of C800 for 24 h. PBS (0.1% diluted in media) was used as a vehicle control. Cell cycle distribution was assessed by flow cytometry analysis and data analyzed by FlowJo. Each experiment was performed in triplicate. The percentage of cells in each phase of the cycle is shown as the mean ± SD and represented as bar diagram. Statistical analysis was performed with one way ANOVA with Tukey’s posthoc test **** *p* < 0.0001, *** *p* < 0.001, ** *p* < 0.01, * *p* < 0.1, ns = not significant.

Disturbances in the cell cycle control are known to inhibit cell growth and activate apoptosis processes [44]. To examine whether C800 affected the cell cycle in TNBC cells, we investigated the percentage of cells in different phases of the cell cycle in T11 and SUM159PT cell populations after treatment with C800 by flow cytometry. Cells were treated with 0.5, 1, 5, 10, and 20 µM of C800 for 24 h, while cells treated with 0.1% PBS were monitored as a vehicle control. T11 and SUM159PT cells treated with colchicine were used as positive controls. Our results revealed that treatment with 5, 10, and 20 µM of C800 caused statistically significant arrest of the cells in the G2/M phase of the cell cycle relative to that of vehicle treated cells, whereas no effect was seen on cells treated with 0.5 and 1 µM C800 (Figure 3A). In T11 cells, at 5, 10, and 20 µM treatment, the percentage of cells at G2/M significantly increased from 24.43% in vehicle control to 49.55%, 38.99%, and 35.53%, respectively, in treated cells (Figure 3A). Similarly, in SUM159PT cells, the percentage of cells at G2/M significantly increased from 31.75% in vehicle control to 48.55%, 44.10%, and 49.30%, respectively, in 5, 10, and 20 μM-treated cells (Figure 3B). Thus, concentrations close to and higher than the IC_50_ arrested the cells at G2/M phase of the cell cycle in TNBC cells.

### 2.4. C800 Down-Regulates Cell Cycle Related Proteins in TNBC Cells

**Figure 4:**
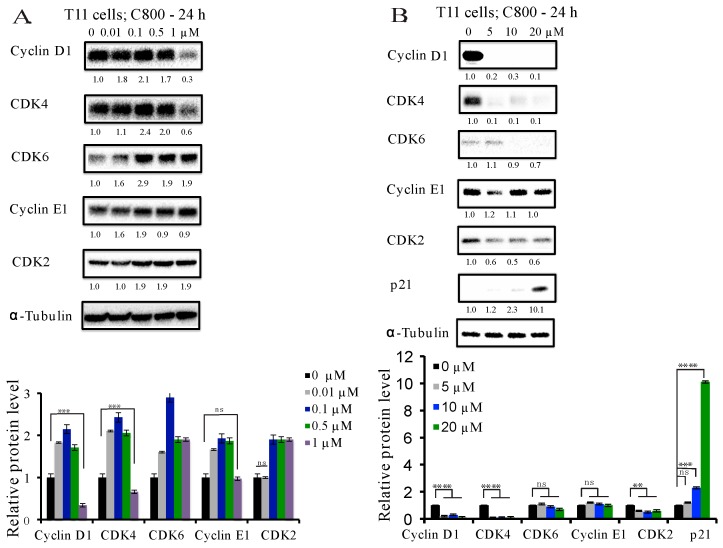

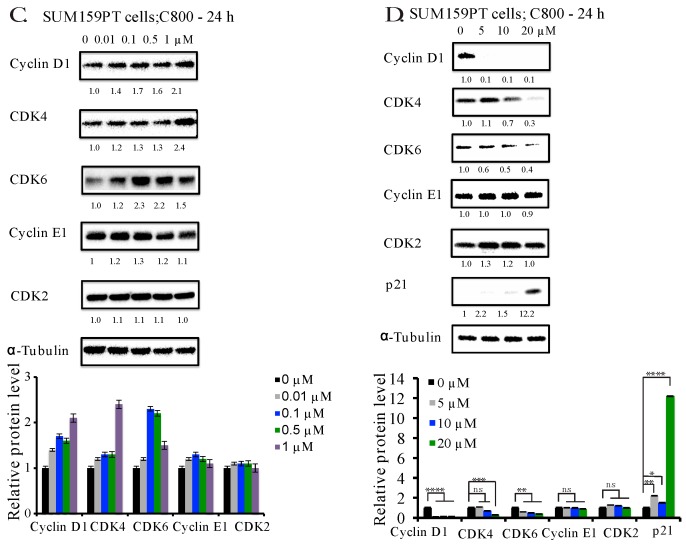
Effect of C800 in the cell cycle-related proteins in TNBC cells. T11 cells were treated with different concentrations of C800 (**A**) 0, 0.01, 0.1, 0.5, and 1 μM; and (**B**) 0, 5, 10, and 20 μM for 24 h. SUM159PT cells were treated with different concentrations of C800 (**C**) 0, 0.01, 0.1, 0.5, and 1 μM; and (**D**) 0, 5, 10, and 20 μM for 24 h. Whole cell lysates were collected. Proteins were quantified and analyzed by immunoblotting with antibodies specific for cyclin D1, CDK4, CDK6, cyclin E1, CDK2, and p21. For each experiment, α-tubulin was used as a loading control. Blots were quantified using ImageJ software (ImageJ 1.50i, NIH, Bethesda, MD, USA), normalized first against loading control (tubulin) and finally against control protein when zero concentration of C800 was added. The data are representative of two independent experiments. The average normalized densities of the band are added below each band and the histograms are presented below the western blots. Statistical analysis was performed with one way ANOVA with Tukey’s posthoc test **** *p* < 0.0001, *** *p* < 0.001, ** *p* < 0.01, * *p* < 0.1, ns = not significant.

In order to explore the mechanism underlying the cell cycle arrest and the decrease in proliferation by C800, we used immunoblotting to investigate the protein expression of well-known key cell cycle checkpoints. Cell cycle progression is known to be positively regulated by cyclins and cyclin-dependent kinases (CDKs), while it is negatively influenced by cyclin-dependent kinase inhibitors (CDKIs), such as p21 [45]. Cyclins play an important role in cell cycle control, several of which represent acknowledged oncogenes [46]. Extensive reports demonstrate that cyclin D1 is an oncogenic driver, amplified and up-regulated in breast carcinoma. Increased expression of cyclin D1 is also associated with resistance to both chemotherapy and hormonal therapy, and, thus, reducing the expression of cyclin D1 is a clinically relevant goal for breast cancer patients [47]. We found that in T11 cells, treatment with C800 at concentrations of 1, 5, 10, and 20 µM decreased the expression of cyclin D1, CDK4, and CDK2, whereas the reduction of CDK6 only occurred at 10 and 20 µM (Figure 4A,B) and the expression of cyclin E1 remained unchanged (Figure 4B). The expression of CDKI, p21 increased only at 20 µM treatment (Figure 4B). Similarly, in SUM159PT cells, the inhibition of the expression of cyclin D1, CDK4, and CDK6 occurred at concentrations higher that 5 µM (Figure 4D). No change was observed in the expression of cyclin E1 and CDK2. Treatment with 20 µM of C800 caused the increase in expression of p21 in SUM159PT cells (Figure 4D). Thus, the decline in cyclin-related proteins correlates with the arrest of the cells in G2/M phase of the cycle.

### 2.5. C800 Downregulates Phosphorylation of AKT/mTOR, MAPK, and NF-κB Signaling Pathways in TNBC Cells

The effect of C800 on down-regulation of cyclin D1 and the proliferation prompted us to investigate the signaling pathways involved in such dysregulation in TNBC cells. TNBC cells are often characterized by up-regulation of phosphatidylinositol-3-kinase (PI3K)/Akt, mammalian target of rapamycin (mTOR), mitogen-activated protein kinase (MAPK), and NF-κB kinases pathways [48,49,50,51]. The (PI3K)/Akt and mTOR signaling pathways represent key regulators of survival during cellular stress and play an important role in survival and chemo-resistance of stem cells such as those found in TNBC [48,52,53]. Notably, an activation of phosphorylation of Akt/mTOR is positively correlated with tumor relapse and metastasis [54]. Similarly, NF-κB pathway is a major regulator of cell survival, proliferation, and differentiation [51]. It has been shown that activation of Akt regulates NF-κB via phosphorylation of p65 by IKB kinase (IKK) both directly and indirectly, thus inhibiting apoptosis [55,56]. Also, MAPK signaling pathways play a fundamental role in cell proliferation, differentiation, survival, and apoptosis, and their aberrant functioning is related to tumorigenesis [57]. It has been demonstrated that PI3k/Akt and MAPK pathways, involving multiple signaling routes, interact and influence each other both positively and negatively, thus demonstrating a crosstalk exists between both of these pathways [58]. Furthermore, cyclin D1 is a direct target of Akt/mTOR, MAPK, and NF-κB signaling pathways [59,60,61], and thus inhibiting these pathways in TNBC has great therapeutic interest.

**Figure 5:**
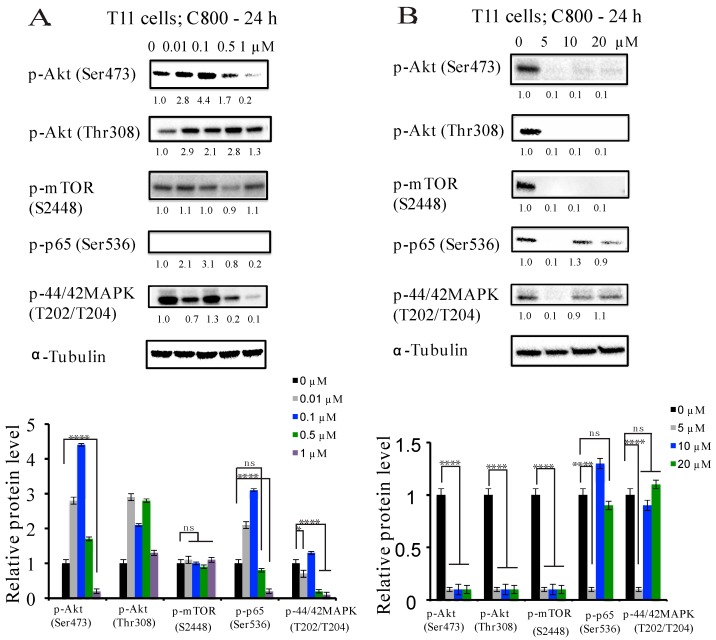

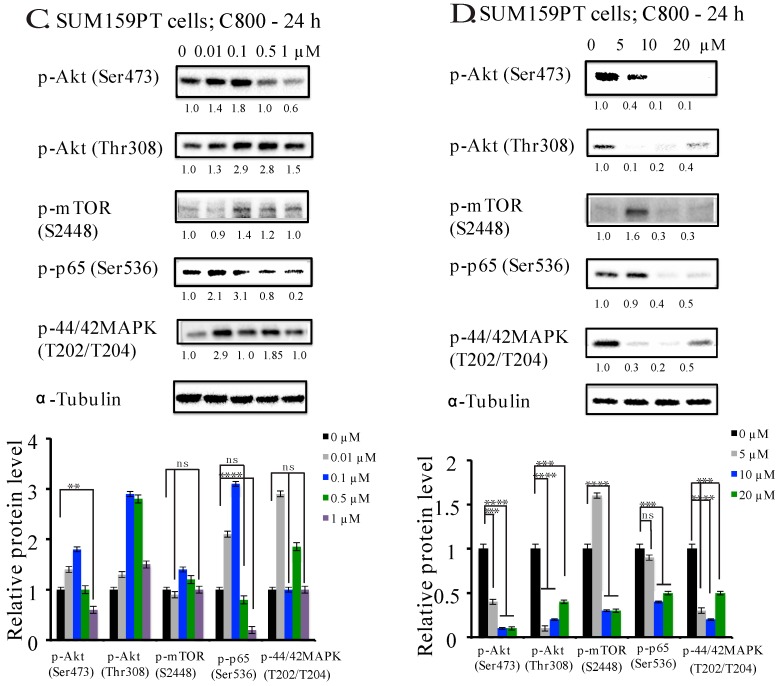
C800 inhibits phosphorylation of Akt, NF-kB, and MAPK pathways in TNBC cells. T11 cells were treated with different concentrations of C800 (**A**) 0, 0.01, 0.1, 0.5, and 1 μM; and (**B**) 0, 5, 10, and 20 μM for 24 h. SUM159PT cells were treated with different concentrations of C800 (**C**) 0, 0.01, 0.1, 0.5, and 1 μM; and (**D**) 0, 5, 10, and 20 μM for 24 h. Whole cell lysates were collected, and proteins were quantified and analyzed by immunoblotting with antibodies specific for p-Akt (Ser473), p-Akt (Thr308), p-mTOR (S2448), p-p44/42 MAPK (T202/T204), and p-p65 (Ser536). For each experiment, α-tubulin was used as a loading control. Blots were quantified using ImageJ software, normalized first against loading control (tubulin) and finally against control protein when zero concentration of C800 was added. The data are representative of two independent experiments. The average normalized densities of the band are added below each band, and the histograms are presented below the western blots. Statistical analysis was performed with one way ANOVA with Tukey’s posthoc test **** *p* < 0.0001, *** *p* < 0.001, ** *p* < 0.01, * *p* < 0.1, ns = not significant.

We first examined the effect of C800 on the phosphorylation of Akt/mTOR, MAPK, and NF-κB pathways in T11 and SUM159PT cells after exposure to different concentrations of C800 for 24 h. At concentrations lower than IC_50_, we found that in T11 cells, 1 µM C800 downregulated the expression of phosphorylation of Akt (Ser473), p65 (Ser536), and p-44/42 (T202/204) (Figure 5A), whereas in SUM159PT cells at 1 µM, the downregulation of phosphorylation of only Akt (Ser473) and p65 was observed (Figure 5C). The expression of phosphorylation of MAPK remained unchanged at lower concentrations compared to vehicle control (Figure 5A,C). However, in both T11 and SUM159PT cells, treatment with concentrations equal to and higher than IC_50_ (5, 10, and 20 µM) completely inhibited phosphorylation of Akt/mTOR, and reduced the expression of phosphorylation of MAPK and p65 compared to vehicle control cells (Figure 5B,D), except in SUM159PT cells, in which the suppression of phosphorylation of mTOR occurs only at 10 µM concentration (Figure 5D). 

**Figure 6:**
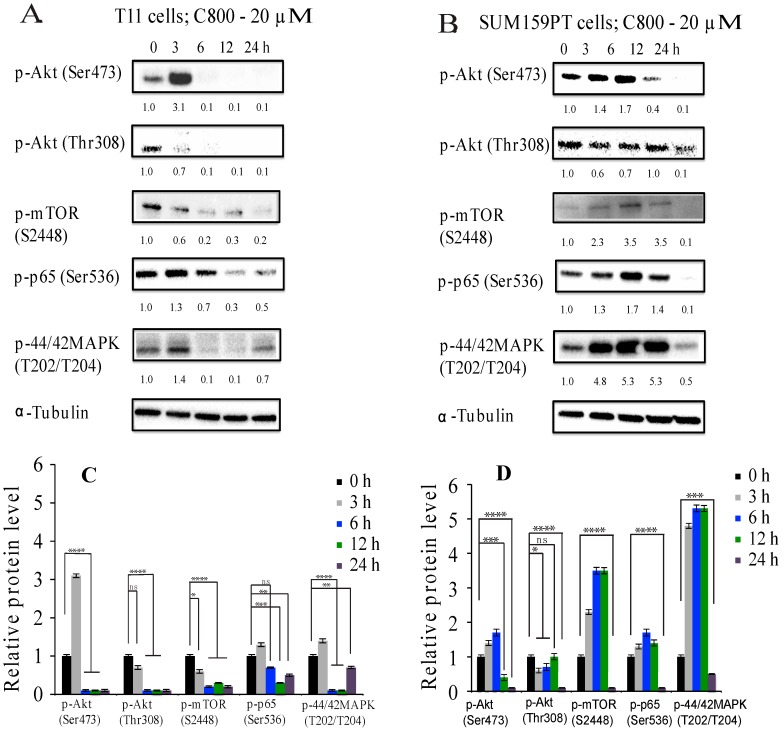
Effect of C800 in inhibition of phosphorylation of Akt, NF-kB, and MAPK pathways in TNBC cells at different time-points. T11 cells were treated with different concentrations of C800 (**A**) 0, 0.01, 0.1, 0.5, and 1 μM; and (**B**) 0, 5, 10 and 20 μM for 24 h. SUM159PT cells were treated with different concentrations of C800 (**C**) 0, 0.01, 0.1, 0.5, and 1 μM; and (**D**) 0, 5, 10, and 20 μM for 24 h. Whole cell lysates were collected, proteins were quantified and analyzed by immunoblotting with antibodies specific for p-Akt (Ser473), p-Akt (Thr308), p-mTOR (S2448), p-p44/42 MAPK (T202/T204), and p-p65 (Ser536). For each experiment, α-tubulin was used as a loading control. Blots were quantified using ImageJ software, normalized first against loading control (tubulin) and finally against control protein when zero concentration of C800 was added. The data are representative of two independent experiments. The average normalized densities of the band are added below each band, and the histograms are presented below the western blots. Statistical analysis was performed with one way ANOVA with Tukey’s posthoc test **** *p* < 0.0001, *** *p* < 0.001, ** *p* < 0.01, * *p* < 0.1, ns = not significant.

To test the effect of C800 further, cells were treated at the highest concentration (i.e., 20 µM) for different times. In T11 cells, we found that the suppression of p-Akt (Ser473), p-p65 (Ser536), and p-p44/42 MAPK (T202/T204) occurred after 6 h of exposure, whereas that of p-mTOR (S2448) and p-Akt (Thr308) was evident after 3 h of treatment (Figure 6A). Moreover, the inhibition of the phosphorylation of mTOR pathway occurred before the inhibition of the Akt pathways. In SUM159PT cells, the inhibition of phosphorylation of Akt (Ser473) pathways occurred after 12 h, whereas that of p-Akt (Thr308), p-p65 (Ser536), p-mTOR (S2448), and p-p44/42 MAPK (T202/T204) occurred only after 24 h of treatment compared to vehicle control (Figure 6B). Moreover, the inhibition of the Akt pathway occurred before the inhibition of the NF-κB, supporting the idea of a temporal crosstalk between these two signaling pathways in SUM159PT cells.

Thus, the inhibition of phosphorylation of these signaling pathways correlates with the arrest of the cells at G2/M phase of the cycle and decline in the cyclin related proteins in TNBC cells. These results suggest that the effect of C800 on cell viability is associated with the arrest of the cells in the G2/M phase of the cell cycle, leading to decline in the cyclin-related proteins and thus inhibition of the phosphorylation of the Akt/mTOR, MAPK, and NF-κB signaling pathways. As Akt/mTOR, MAPK, and NF-κB signaling pathways are considered the key targets for TNBC, identifying novel and potent inhibitors concomitantly targeting these pathways would be useful in treatment of TNBC by decreasing potential escape mechanisms. Overall, our results suggest that C800 could potentially be used for targeting multiple signaling pathways involved in cell survival and resistance to chemotherapy. 

### 2.6. C800 Induces Apoptotic Cell Death in TNBC Cells

**Figure 7:**
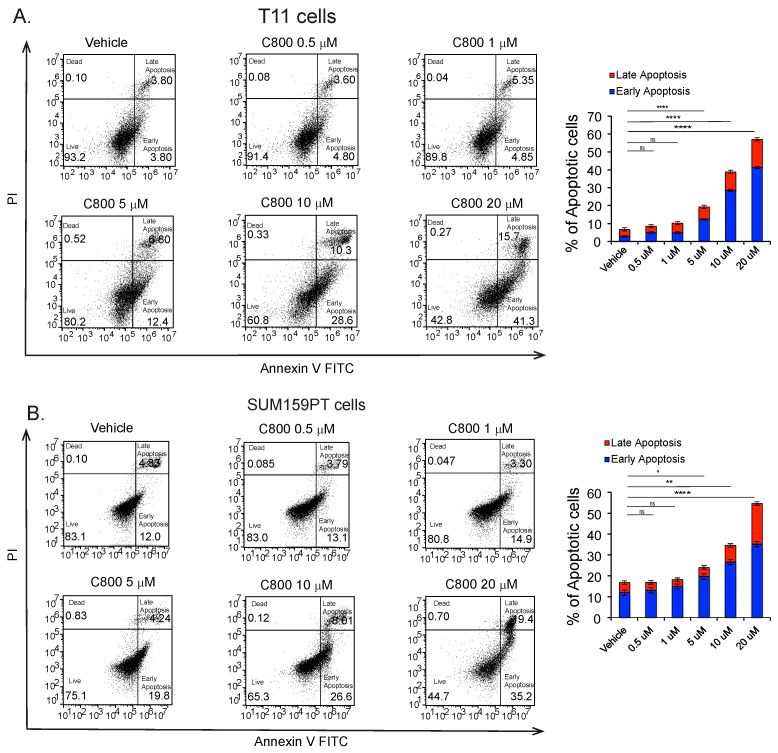
C800 induces apoptosis in TNBC cells. (**A**) T11 cells and (**B**) SUM159 cells were treated with vehicle (0.1% PBS diluted in media) and different concentrations of C800 (0.5, 1, 5, 10, and 20 μM) for 24 h. Flow cytometry analysis of T11 and SUM159PT cells when treated with C800 was performed by double staining using Annexin-V FITC and PI, and data were analyzed using Flow Jo 10. Experiments were done in triplicate and the results were plotted for percentage of apoptotic cells (early and late apoptosis appearing in the right lower and upper quadrants) as bar diagram and represented as mean ± S.D. Statistical analysis was performed with one way ANOVA with Tukey’s posthoc test **** *p* < 0.0001, *** *p* < 0.001, ** *p* < 0.01, * *p* < 0.1, ns = not significant.

Apoptosis or programmed cell death is a cellular event that occurs during DNA repair and tissue remodeling during early development. Here, we investigated whether C800 induced apoptosis in TNBC cells. T11 and SUM159PT cells were treated with different concentrations (0, 0.5 1, 5, 10, 20 µM) of C800 for 24 h, and the percentage of apoptotic cells were evaluated by flow cytometry by double staining using Annexin-V FITC and Propidium Iodide. Cells treated with doxorubicin were used as positive controls. We found that when treated at 0.5, 1, 5, 10, and 20 µM, the total percentage of cells (early and late apoptotic population) increased from 6.7% in control group to 8.5%, 10.2%, 19.2%, 38.9%, and 57.0%, respectively, in T11 cells and from 16.0% in control group to 17.1%, 18.3%, 24.0%, 34.3%, and 50.4%, respectively, in SUM159PT cells (Figure 7B). Thus, these results suggest that C800 caused apoptotic cell death in TNBC cells.

**Figure 8:**
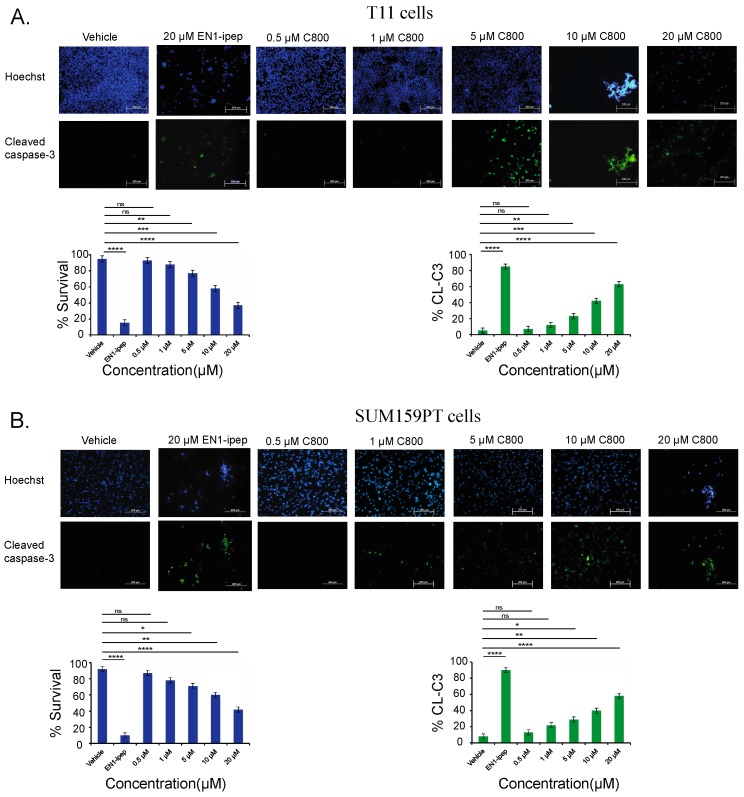
C800 induces apoptosis in TNBC cells. (**A**) T11 cells and (**B**) SUM159 cells were treated with vehicle (0.1% PBS diluted in media), 20 μM of EN1-ipep (positive control), and different concentrations of C800 (0.5, 1, 5, 10, 20 μM) for 24 h and then processed for immunofluorescence assay. Green nuclei represent cleaved-caspase-3 positive cells. Nuclei were counterstained with Hoechst 33258. Blue and green positive cells were quantified using ImageJ (ImageJ 1.50i, NIH, Bethesda, MD, USA), software and represented graphically by bar diagram. Experiments were done in triplicate and the results represent mean ± S.D. Statistical analysis was performed with one way ANOVA with Tukey’s posthoc test **** *p* < 0.0001, *** *p* < 0.001, ** *p* < 0.01, * *p* < 0.1, ns = not significant.

Another key feature of apoptosis is the cleavage and consequent activation of the executor, caspase-3, which in turn initiates the apoptotic cascade by substrate cleavage, leading to programmed cell death. Therefore, cleaved caspase-3 is widely used as a marker for apoptosis [62,63]. To further verify the percentage of apoptotic cells in TNBC models, T11 and SUM159PT cells were treated with 0.5, 1, 5, 10, and 20 µM of C800 for 24 h. Cells treated with 20 µM of EN1-ipep (nanoparticles conjugated with interference peptide for homeobox transcriptional factor Engrailed, which induces apoptosis in basal-like breast cancer cells) were used as positive control [32]. After the treatments, cell apoptosis was assessed by immunofluorescence using an anti-cleaved caspase-3 antibody. Percentage of apoptosis was compared to that of vehicle control treated cells. In T11 cells, treatment with 0.5, 1, 5, 10, and 20 µM increased the apoptotic cell population from 5% in control group to 7%, 12%, 23%, 42%, and 63%, respectively, (Figure 8A), and from 8% in control group to 13%, 22%, 29%, 40%, and 58%, respectively, in SUM159PT cells (Figure 8B). Thus, we found that treatment with C800 resulted in dose-dependent apoptotic cells in the T11 and SUM159PT cells. The result was consistent with the one obtained from Annexin V/PI experiment.

## 3. Materials and Methods

### 3.1. Experimental Procedures

All NMR spectra were recorded on Bruker Avance 600 MHz spectrometer (Bruker Corporation, Karlsruhe, Germany) using methanol-d_4_ as the solvent. Chemical shifts were reported with reference to the residual solvent peak δ_H_ 3.31 and δ_C_ 49.0 ppm. High-resolution mass spectra were recorded on Waters LCT Premier XE (Waters, Sydney, NSW, Australia) time-of-flight mass spectrometer in positive electrospray ionization mode by direct infusion of the purified compounds. Semi-preparative HPLC was performed using an Agilent Series 1200 instrument equipped with a photodiode array detector and preparative fraction collector. Separation was achieved using an Apollo reversed phase C18 column (250 mm × 10 mm, 5 µm, Grace-Davison Discovery Sciences, Melbourne, VIC, Australia).

### 3.2. Reagents

All materials were purchased from Sigma-Aldrich (St Louis, MO, USA) unless otherwise stated. 

### 3.3. Details of Collection of Sponge Materials and Identification

All specimens were collected in 2005 off the coast of Western Australia and have been stored wet frozen. Further details of the sponge materials used in our study are presented in Table S1 in supplementary material.

### 3.4. Purification and Isolation of Crambescidin 800 (C800)

The wet sponges (240 g) were cut into pieces and extracted thrice with 1:1 dichloromethane (DCM) and methanol overnight at room temperature. The crude extracts were combined and dried under reduced pressure to give a brown oily extract (19.2 g). The crude extract was partitioned between DCM and water to afford the DCM fraction that was dried under reduced pressure to yield a brown solid (7.6 g). The DCM fraction was dissolved in methanol (6 mL) and subjected to flash chromatography using C18 reversed phase silica (Grace Davisil^®^ (Grace, Melbourne, VIC, Australia), C18 silica, pore size 60 Å, particle size 35–70 μm) as the stationary phase. The column was eluted with a stepwise gradient of solvents starting with 100% water and with increasing concentrations of methanol and, finally, 100% methanol to afford six different fractions. These fractions were tested for cell viability in T11 cells (Cell Titer Glo^®^ assay (Promega, Sydney, NSW, Australia)) after 24 h of exposure. Testing of subsets of the fractions showed activity in the 80% methanol fraction. The 80% methanol fraction (0.38 g) was separated further using semi-preparative reversed phase HPLC using an isocratic mobile phase consisting of 45% (*v*/*v*) acetonitrile/water (+ 0.1% TFA) over 40 min. A total of fifteen fractions (40 × 1 min fractions, combined based on peaks observed) were collected and tested in T11 cells. Fraction 5 was identified as the most active fraction, corresponding to a retention time of between 11 and 12 min. Removal of the solvent under reduced pressure yielded C800 as a colorless glassy solid (9 mg, 0.01%, based on the wet weight of the sponge) that was active in T11 cells. Spectroscopic data (below) was consistent with data previously reported for C800 [29,34].

**Crambescidin 800:** colorless glassy solid; ^1^H NMR (CD_3_OD, 600 MHz) δ 5.57 (1H, t, *J* = 8.8 Hz, H-5), 5.51 (1H, d, *J* = 10.9 Hz, H-4), 4.40 (1H, br, d, *J* = 9.3 Hz, H-3), 4.33–4.36 (1H, m, H-10), 4.10–4.15 (2H, m, H-23), 4.04–4.06 (1H, m, H-19), 3.93 (1H, m, H-43), 3.82–3.84 (1H, m, H-13), 3.64–3.69 (1H, m, H-39b), 3.40–3.45 (1H, m, H-42b), 3.37–3.40 (1H, m, H-39a), 3.27–3.28 (1H, m, H-42a), 3.09–3.14 (2H, m, H-45), 3.07 (1H, d, *J* = 5.1 Hz, H-14), 2.96 (1H, t, H-41a), 2.87–2.89 (1H, m, H-41b), 2.65 (1H, dd, *J* = 4.8 Hz, H-9b), 2.43–2.50 (4H, m, H-7b, H-12b, H-37), 2.34–2.40 (2H, m, H-6b, H-11b), 2.28–2.34 (1H, m, H-6a), 2.14–2.18 (1H, m, H-7a), 1.95–1.99 (2H, m, H-12a, H-17b), 1.90–1.91 (4H, m, H-16b, H-40, H-44b), 1.81–1.82 (2H, m, H-18b, H-44a), 1.68–1.73 (2H, m, H-17a, H-18a), 1.60–1.65 (4H, m, H-11a, H-16a, H-24 ), 1.52–1.58 (1H, m, H-2b), 1.41–1.48 (2H, m, H-2a, H-9a), 1.08 (3H, d, *J* = 6.2 Hz, H-20), 0.85 (3H, t, *J* = 7.3Hz, H-1); ^13^C NMR (CD_3_OD, 600 MHz) δ 177.5 (C, C-38), 170.1 (C, C-22), 150.2 (C, C-21), 134.3 (CH, C4), 131.2 (CH, C5), 85.1 (C, C-8), 82.1 (C, C-15), 72.6 (CH, C-3), 69.3 (CH, C-19), 68.3 (CH, C-43), 66.5 (CH_2_, C-23), 55.5 (CH, C-10), 54.1 (CH_2_, C-42), 53.3 (CH, C-13), 47.8 (CH, C-14), 43.8 (CH_2_, C-39), 38.4 (CH_2_, C-9, C-45), 38.1 (CH_2_, C-7), 38.09 (CH_2_, C-41), 37.85 (CH_2_, C-37), 34.1 (CH_2_, C-16), 33.9 (CH_2_, C-44), 32.9 (CH_2_, C-18), 32.6 (CH_2_, C-11), 30.8 (CH_2_, C-24), 30.7 (CH2, C-2), 30.5 (CH_2_, C-12), 25.6 (CH_2_, C-36), 25.3 (CH_2_, C-40), 24.9 (CH_2_, C-6), 20.4 (CH_3_, C-20), 18.1 (CH_2_, C-17), 10.7 (CH_3_, C-1); HRMS (ESI) *m*/*z* 801.6216 [M + H]^+^ (calcd. for C_45_H_81_N_6_O_6_, 801.6218).

### 3.5. Cell Culture

T11 cell line, derived from a murine basal-like breast tumor of a p53^−/−^ transgenic mouse [4,32,33] and human breast cell lines MCF7, SUM159PT, and SUM149PT, was obtained from the Tissue Culture Facility of the UNC Lineberger Comprehensive Cancer Centre (University of North Carolina, Chapel Hill, NC, USA). MDA-MB-231, ZR-75-1, 3T3, and HDF cell lines were purchased from the American Type Culture Collection (Manassas, VA, USA). T11, ZR-75-1 cells were cultured in RPMI medium. SUM159PT and SUM149PT cells were cultured in DMEM-F/12 (Life Technologies; Melbourne, VIC, Australia) media. MDA-MB-231, 3T3, and HDF cells were cultured in DMEM (Life Technologies, Melbourne, VIC, Australia) media. MCF7 cells were cultured in MEM alpha. All media were supplemented with 10% fetal bovine serum (Life Technologies, Melbourne, VIC, Australia) and 1% penicillin/streptomycin (Life Technologies, Melbourne, VIC, Australia), except SUM159PT media that was supplemented with 5% fetal bovine serum. MCF7 media was supplemented with 1% sodium bicarbonate (100 mM), 1% sodium pyruvate (7.5 mM), and 1% MEM non-essential amino acid (100×) (Life Technologies, Melbourne, VIC, Australia). SUM159PT media was supplemented with 5 μg/mL insulin and 1 μg/mL hydrocortisone. Cell lines were cultured in 10 cm^2^ petri dish (Corning) and were maintained in humidified incubators at 37 °C with 5% CO_2_. Cells were passaged at 80% confluency, and media changed every 4–5 days.

### 3.6. Cell Viability Assay

Cells were plated in 96-well white plate at a seeding density of 3000 cells/well for T11, 5000 cells/well for SUM159PT and MDA-MB-231, 7000 cells/well for MCF7 and SUM149PT, and 8000 cells/well for HDF, 3T3, and ZR-75-1, respectively. The cells were left to adhere overnight. As the crude extract was only soluble in DMSO, the extract before separation was dissolved in 1% DMSO for testing in the cells. After separation and purification, C800 was soluble in PBS, hence PBS was used to dissolve C800. Purified C800 was dissolved in PBS and diluted with media to get the required concentrations. Cells were treated with C800 for 24 h. Cell viability was determined using Cell Titer Glo^®^ according to manufacturer’s protocol (Promega; Sydney, NSW, Australia) and luminescence was measured using the Envision 2012 Multi-label Reader (PerkinElmer; Waltham, MA, USA). IC_50_ of C800 in various cell types was calculated using Graphpad Prism 6.

### 3.7. Cell Cycle Analysis

Cells were plated in 6 well plates at a seeding density of 500,000 cells/well and left to adhere overnight. The next day, cells were treated with C800 with various concentrations for 24 h. Cells were harvested, washed with FACS buffer (2% FBS in PBS), and then fixed with 70% ethanol in de-ionised water at −20 °C overnight. The fixed cells were washed with cold PBS and incubated for 30 min at 37 °C with RNase A (50 ug/mL, Qiagen Kit (Melbourne, VIC, Australia)). Finally, cells were stained with Propidium iodide (50 µg/mL, Sigma Aldrich (St Louis, MO, USA)) for 30 min at room temperature. The stained cells were analyzed by flow cytometry (BD Accuri C6). Data were analyzed with FlowJo 10 (FlowJo, Ashland, OR, USA).

### 3.8. Western Blot Analysis

Cells were plated in 6 well plates at a seeding density of 1,000,000 cells/well and treated with various concentration of C800 at different time points. Cells were harvested and lysed on ice for 5 min in lysis buffer (400 mM NaCl, 0.5% triton X-100, 50 mM tris pH 7.4) and sonicated for 40 s at 10 mA. Proteins were quantified and equal amount of proteins from each group were separated in 4–15% SDS-PAGE (BioRad (Sydney, NSW, Australia)) and transferred electrically onto PVDF membrane (BioRad (Sydney, NSW, Australia)). The membrane was blocked with 5% skim milk powder in TBST, washed thrice with TBST, and incubated with specific primary antibodies overnight at 4 °C. The membranes were blotted for cyclin D1, cyclin E1, CDK4, CDK6, CDK2, p21, p-Akt (Ser473), p-Akt (Thr308), p-mTOR (Ser2448), p-p44/42 MAPK (T202/T204), and p-p65 (Ser536) (Cell Signaling Technologies, Brisbane, QLD, Australia). Dilutions were made according to manufacturer’s protocol. Then, the membrane was washed thrice with TBST and incubated with horseradish peroxidase (HRP)-conjugated secondary antibodies (1:10,000 dilution, GE Technologies, Brisbane, QLD, Australia) for 1 h at room temperature. The proteins were detected using Clarity Western ECL detection kit (BioRad (Sydney, NSW, Australia)). α-Tubulin (monoclonal, 1:5000, clone B512, Sigma-Aldrich, St Louis, MO, USA) was used as a loading control.

### 3.9. Apoptosis Assay (Annexin-V-PT-Binding Assay)

To determine whether C800 affected the apoptosis of TNBC cells, the apoptotic rate of TNBC cells was detected using Annexin-V/FITC Apoptosis Detection Kit according to manufacturer’s protocol (BD Bioscience (Perth, WA, Australia). Cells were plated in 6 well plates at a seeding density of 500,000 cells/well and left to adhere overnight. The next day, cells were treated with C800 with various concentrations for 24 h. Cells were harvested, washed twice with ice-cold PBS, and dissolved in 1× binding buffer. Finally 100,000 cells were stained with Annexin-V/FITC and Propidium iodide for 15 min at room temperature in the dark. The stained cells were diluted with 1× binding buffer and analyzed by flow cytometry (BD Accuri C6, Sydney, NSW, Australia). Data were analyzed with FlowJo 10 (FlowJo LLC, Ashland, OR, USA).

### 3.10. Apoptosis Assay (Immunofluorescence)

Cells were plated in coverslips pre-coated with poly-lysine, in 24 well plated at a seeding density of 100,000 cells/well, and left to adhere overnight. The cells were treated with C800 for 24 h. After 24 h, cells were fixed with 4% paraformaldehyde for 20 min at room temperature, washed thrice with PBS, blocked with blocking solution (3% BSA in PBS), and incubated with an anti-cleaved caspase-3 primary antibody (1:500 dilution, Cell Signaling Technology, Brisbane, QLD, Australia) overnight at 4 °C. The next day, the cells were washed thrice with PBS and incubated with an anti-rabbit secondary antibody Alexa Fluoro 488-conjugated antibody (1:5000 dilution; Cell Signaling Technologies, Brisbane, QLD, Australia) and Hoechst 33258 (1:10,000 dilution) nuclei for 1 h at room temperature. The coverslips were washed thrice with PBS and mounted on slides. The images were recorded in Olympus IX71 microscope (Melbourne, VIC, Australia). 

### 3.11. Statistical Analysis

Statistical analyses were performed with GraphPad Prism version 6 (GraphPad Software Inc., La Jolla, CA, USA). Statistical significance was determined using an unpaired one-way ANOVA with the Tukey’s posthoc test correcting for multiple comparisons and Student’s *t*-test.

## 4. Conclusions

In the present work, we isolated crambescidin 800 from the marine sponge, *Monanchora viridis*, collected in Western Australia, and demonstrated the enhanced cytotoxic effect of this guanidine alkaloid in triple negative breast cancer (TNBC) cell lines. C800 reduced cell viability and caused cell cycle arrest at G2/M phase in T11 and SUM159PT cells. In addition, C800 reduced the expression of CDKs responsible for proliferation such as cyclin D1, CDK4, and CDK6, and enhanced the expression of CDKIs such as p21, at the protein level. Importantly, C800 inhibited the phosphorylation of the Akt/mTOR, MAPK, and NF-κB pathways, which are responsible for tumor relapse and metastasis, leading to programmed cell death in T11 and SUM159PT TNBC cells. Overall, our results show that C800 could potentially be used for targeting multiple signaling pathways in TNBC cells, known to be involved in cell survival and resistance mechanisms to chemotherapy. Further studies are needed to test whether C800 has in vivo activity against TNBC and thus any potential anticancer therapy.

## Supplementary Materials

The following are available online at http://www.mdpi.com/1660-3397/16/2/53/s1, ^1^H and ^13^C NMR spectra of isolated crambescidin 800 and the details of the sponge materials used in this study.
