# Supplementary material for: Crambescidin 800, Isolated from the Marine Sponge Monanchora viridis, Induces Cell Cycle Arrest and Apoptosis in Triple-Negative Breast Cancer Cells
Source: Mar Drugs. 2018 Feb 8;16(2):53. doi: 10.3390/md16020053 (PMC5852481; doi:10.3390/md16020053)
Supplement: Supplementary file 1 [file marinedrugs-16-00053-s001.docx]

Supporting Information

Crambescidin 800, isolated from the marine sponge *Monanchora viridis*, induces cell cycle arrest and apoptosis in Triple-Negative Breast Cancer cells

Sumi Shrestha^1,2^, Anabel Sorolla^2^, Jane Fromont^3^, Pilar Blancafort^2^*, and Gavin R. Flematti^1^*

^1^ School of Molecular Sciences, The University of Western Australia, Crawley, Australia; [sumi.shrestha@research.uwa.edu.au](mailto:sumi.shrestha@research.uwa.edu.au) (S.S)

^2^ Cancer Epigenetics, Harry Perkins Institute of Medical Research, Nedlands, Australia; [anabel.sorollabardaji@uwa.edu.au](mailto:anabel.sorollabardaji@uwa.edu.au) (A.S)

^3^ Western Australian Museum, Welshpool, Australia; [jane.fromont@museum.wa.gov.au](mailto:jane.fromont@museum.wa.gov.au) (J.F)

* Correspondence: [gavin.flematti@uwa.edu.au](mailto:gavin.flematti@uwa.edu.au); Tel.: +61 6488 1005 (Chemistry) (G.R.F)

[pilar.blancafort@uwa.edu.au](mailto:pilar.blancafort@uwa.edu.au); Tel.: +61 8615 1099 (Cancer Biology) (P.B)

Academic Editor: name

Received: date; Accepted: date; Published: date

**Contents:**

**T1.** Details of sponge specimens tested in this study

1. ^1^H NMR of Crambescidin 800 (C800) in CD3OD (600 MHz)
2. ^13^C NMR of Crambescidin 800 (C800) in CD3OD (600 MHz)

**Table 1:** Details of sponge specimens tested in this study

| **Museum registration number** | **Sponge identification** | **Collection location (WA)** | **Depth (metres)** |
| --- | --- | --- | --- |
| Z35820 | *Monanchora viridis* | Off Cape Mentelle, South-west WA | 97 |
| Z36271  Z31539  Z35794  Z35818  Z36203  Z36256  Z35788  Z36251  Z35766  Z35949  Z35252  Z35255  Z35298  Z35633  Z35792  Z35798  Z35819  Z36242  Z36244 | *Monanchora* sp. nov*.*  *Manihinea lynbeazleyae*  *Jaspis* SS5  *Halichondria* (*Halichondria*) SS5  *Sarcotragus* SS13  *Hippospongia* SS3  *Biemna* SS2  *Hemiasterella* SS2  *Mycale (Carmia)* SS3  *Ecionemia* SS1  *Cinachyrella* SS2  *Coelosphaera* (*Coelosphaera*) SS3  *Niphates* SS2  *Dactylospongia* SS1  *Clathria* (*Thalysias*) SS1  *Spongia* (*Heterofibria*) SS1  *Hyrtios* SS4  *Agelas* SS3  *Callyspongia* (*Callyspongia) bilamellata* | Off Carnarvon  Perth Canyon  Off Cape Mentelle  Off Albany  Imperieuse  Off Albany  Off Bald Island  Off D’Entrecasteaux  Off Albany  Off Zuytdorp  Perth Canyon  Perth Canyon  Perth Canyon  Perth Canyon  Off Albany  Off Albany  Houtman Abrolhos  Off Cape Mentelle  Off Cape Mentelle | 196  232  97  100  80  100  99  100  100  106  232  232  232  232  212  100  180  97  9 |
